# High-Value Care in the Evaluation of Stroke

**DOI:** 10.7759/cureus.1532

**Published:** 2017-08-01

**Authors:** Prakrity Urja, Eric H Nippoldt, Virginia Barak, Carrie Valenta

**Affiliations:** 1 Creighton University Medical Center, CHI Creighton University; 2 School of Medicine, Creighton University School of Medicine

**Keywords:** value-based care, high value care, embolic stroke

## Abstract

Value-based care emphasizes achieving the greatest overall health benefit for every dollar spent. We present an interesting case of stroke, which made us consider how frequently health care providers are utilizing value-based care.

A 73-year-old Caucasian, who was initially admitted for a hypertensive emergency, was transferred to our facility for worsening slurring of speech and left-sided weakness. The patient had an extensive chronic cerebrovascular disease, including multiple embolic type strokes, mainly in the distribution of the right temporal-occipital cerebral artery and transient ischemic attacks (TIAs). The patient had a known history of patent foramen ovale (PFO) and occlusion of the right internal carotid artery. He was complicated by intracranial hemorrhage while on anticoagulation for pulmonary embolism. He was chronically on dual antiplatelet therapy (aspirin and clopidogrel) and statin.

Following the transfer, stroke protocol, including the activation of the stroke team, a computed tomography (CT) imaging study, and the rapid stabilization of the patient was initiated. His vitals were stable, and the physical examination was significant for the drooping of the left angle of the mouth, a nonreactive right pupil consistent with the previous stroke, a decreased strength in the left upper and lower extremities, and a faint systolic murmur.

His previous stroke was shown to be embolic, involving both the right temporal and occipital regions, which was re-demonstrated in a CT scan. A magnetic resonance imaging (MRI) scan of the brain showed a new, restricted diffusion in the right pons that was compatible with an acute stroke as well as diffusely atherosclerotic vessels with a focal stenosis of the branch vessels. A transthoracic echocardiogram demonstrated no new thrombus in the heart. A transesophageal echocardiogram (TEE) showed known PFO, and repeat hypercoagulation evaluation was negative, as it was in his previous cerebrovascular accident (CVA) evaluation.

Appropriate medical treatment with antiplatelets, as indicated by the acute stroke guidelines, was started. The patient was not eligible for thrombolysis.

Value-based care emphasizes the decreased usage in investigations or health care of options that do not contribute to the overall health and well-being of the patient.

Given our patient's past medical history and the results of previous investigations, we questioned the value of ordering a hypercoagulable evaluation and TEE in our patient. The need for an evaluation of the hypercoagulable state in an elderly patient with ischemic stroke or TIA remains unknown. Our patient had a complete hypercoagulable evaluation done six years earlier. Repeating the hypercoagulable evaluation would not contribute to the treatment decisions and, as a result, would not satisfy the basic criteria for value-based care.The importance of a repeat TEE is uncertain in the evaluation of embolism for a known cause of stroke. Additionally, no change in management was anticipated regardless of the TEE findings, therefore, repeating TEE in our patient was an inappropriate use of resources.

Being mindful of value-based care can reduce overall health care costs, maintain our role of being responsible stewards of our limited resources, and continue to provide high-value care for our patients.

## Introduction

Value-based care, which is being increasingly promoted on a global scale, emphasizes achieving the maximum health benefit for every dollar spent. Value-based care does not advocate saving by means of cost shifting or by restricting services [1]. Instead, it emphasizes a decrease in investigations or health care services that do not contribute to the overall best care of the patient. Stroke is one of the most common causes of admission to the hospital. The care of a stroke patient imposes a significant economic burden to both the individual and the overall health care system [2]. The average annual cost of inpatient stroke care in the United States in 2012 was $ 33 billion, and it is projected to increase by three times by the year 2030 [2]. We present a case of a patient who underwent a stroke evaluation that prompts us to consider, from the perspective of care quality, cost effectiveness, and benefits, how frequently we use value-based care in practice.

## Case presentation

A 73-year-old Caucasian male presented to an outside hospital for worsening slurring of speech, near syncope, dizziness, and left-sided weakness of one-day duration. On presentation, he was found to have a significantly elevated blood pressure of 231/124. However, no new neurologic deficits were found, and the workup was grossly normal except a re-demonstration of the right temporal and the occipital lesion on a computed tomography (CT) scan of the head from his previous stroke. The patient was subsequently admitted to the intensive care unit (ICU) on a nicardipine drip for the treatment of the hypertensive emergency and, as recommended, a blood pressure drop of less than 25% was achieved in 24 hours.

The next morning, the patient developed worsening slurred speech, right-sided facial droop, left-sided, upper-extremity weakness, and confusion. A repeat CT scan of the head showed no acute events. He was then transferred to our facility for further evaluation and treatment.

The patient had an extensive chronic cerebrovascular disease, including multiple embolic type strokes, mainly in the distribution of the right temporal-occipital cerebral artery and transient ischemic attacks (TIAs). The patient had a known history of patent foramen ovale (PFO) and the occlusion of the right internal carotid artery. He was complicated by intracranial hemorrhage while on anticoagulation for pulmonary embolism. He was chronically on dual antiplatelet (aspirin and clopidogrel) and atorvastatin. His other medical condition included mitral valve prolapse (MVP), essential hypertension, hyperlipidemia, bilateral hilar, and mediastinal lymphadenopathy of unknown significance.

On presentation to our facility, his vitals were stable with blood pressure: 146/69 mmHg, heart rate: 69 beats/min, respiratory rate: 18 breaths/min, and temperature: 36.6 C (97.9 F).

The patient appeared in no acute distress and was oriented to person, place, and time. The left angle of his mouth drooped, drooling was present, and he demonstrated a left upper motor seventh nerve palsy. He also demonstrated a nonreactive right pupil, normal accommodation, and a loss of sight in the right eye consistent with his prior CVA. He exhibited increased muscle tone and decreased strength in the left upper and lower extremity with a relatively weaker grip as compared to the right side. On cardiac examination, he had a normal rate, a regular rhythm, and a faint systolic murmur heard at the apex, consistent with his history of MVP. There was no edema in the lower extremities. He had normal respiratory effort and lungs were clear to auscultation bilaterally. The rest of the examination was unremarkable. He had a sinus rhythm with mild left ventricular hypertrophy in the electrocardiogram (EKG) finding on admission.

A neurologist was consulted immediately upon arrival to our hospital, and stroke protocol was initiated. He scored nine on the National Institute of Health Stroke Scale (NIHSS). A CT of the head without contrast was negative for evidence of acute intracranial abnormality, but evidence of a remote infarct of the right temporal and occipital lobe and chronic microvascular ischemia with atrophy and senescent change was present. He was not a candidate for thrombolytic therapy, as his symptoms had been present for greater than 4.5 hours.

He was continued on atorvastatin and started on secondary prevention for stroke - aspirin/dipyridamole (Aggrenox) - since he had previously failed therapy with aspirin and clopidogrel.

His laboratory findings were a creatinine level of 1.38 mg/dl (baseline 1.2 mg/dl) and a glomerular filtration rate of 50 ml/min (baseline: 53 ml/min). The complete metabolic panel was otherwise within normal limits. A complete blood count (CBC) revealed hemoglobin: 12.5 gm/dl (14.5 gm/dl on the last admission), hematocrit: 38.6% (45.9% on the last admission), platelet count: 138 k/ul (208 k/ul on the last admission), and troponin I: <0.04 ng/mg. The remaining values were within normal limits. Hypercoagulable studies were negative.

As a second level of investigation, he received a magnetic resonance imaging (MRI)/magnetic resonance angiogram (MRA) scan, which showed the area of restricted diffusion in the right pons compatible with acute stroke as well as diffusely atherosclerotic vessels with a focal stenosis of branch vessels. An ultrasound doppler of the carotids showed near occlusion of the right internal carotid artery and no evidence of significant stenosis in the left internal carotid artery. All the above imaging was consistent with previous studies. A transthoracic echocardiography (TTE) showed no thrombi with preserved ejection fraction. A transesophageal echocardiography (TEE) showed a ventricular ejection fraction of 50-55% with moderate left atrial enlargement, and no masses or thrombi were noted. However, a small PFO was found on bubble study.

Once deemed stable, the patient was discharged home on atorvastatin and aspirin/dipyridamole (Aggrenox) with discontinuation of his home medication, clopidogrel. He was enrolled in outpatient physical and occupational therapy. A Holter monitor and follow-up with primary care and neurology were arranged.

During his previous right temporal-occipital embolic CVA six years earlier, he had received a thorough embolic and hypercoagulable workup. At that time, the hypercoagulable workup was negative, and TEE was significant for small PFO for which cardiology was consulted and no further treatment was recommended.

## Discussion

Stroke is an important medical problem. Among the stroke subtypes, the majority are ischemic. Ischemia of the brain can be provoked via three mechanisms: thrombosis, embolism, and systemic hypoperfusion. An ischemic stroke can be further divided into large vessel atherosclerotic stenosis, small artery disease (lacunae), cryptogenic and cardiogenic embolism, and unusual causes (e.g., dissection or arteritis).

A cryptogenic stroke in which the cause of the stroke cannot be determined after the exclusion of small vessel disease (lacunae), large artery occlusive disease, and cardiac emboli [3] accounts for 25% of the total cases of ischemic stroke [4]. The diagnosis, in particular, of a cryptogenic stroke can be challenging and often requires an extensive and multilevel diagnostic workup that translates to increased health care cost [5].

Our patient had two episodes of ischemic stroke. His first episode six years prior resulted from an embolic cause. Due to both the lack of a known embolic source as well as the high risk of bleeding while on anticoagulation, the patient was not started on anticoagulation at that time. During this subsequent episode, he was re-evaluated for an embolic source of stroke. His evaluation included echocardiography (both TTE and TEE), inpatient cardiac telemetry, MRI brain, CT brain, MRA brain and neck, and disorders of coagulation.

The current, commonly practiced standard evaluation of stroke include a CT head/MRI brain scan to locate the territory of the ischemia, a CT angiogram/MRA of the neck, a 24-hour Holter monitor, an echocardiogram, a hematological evaluation with red blood cell (RBC) count, platelet count, partial thromboplastin time, and prothrombin time [5-7].

Imaging using ultrasounds, MRIs, and CTs provides clues for different causes of ischemic stroke. An embolic stroke can present as infarcts in multiple territories from the aorto-cardiac origin or multiple infarcts of different ages in the same arterial territory [7]. Systemic hypoperfusion or multiple emboli can present as infarcts between the brain artery territories [7]. In our patient, the MRI scan showed diffusely atherosclerotic vessels with focal stenosis of branch vessels. This finding is highly suggestive of large artery atherosclerosis as the cause of stroke, particularly with the known risk factors of age, hypertension, and hyperlipidemia.

TEE is both an expensive and invasive procedure and can have many complications [8]. Identification of the source of the embolic phenomenon remains the most common indication for TEE following CVA. TEE is recommended to identify valvular pathology, left appendageal thrombosis, thoracic aorta plaque, or thrombus not identified by the TTE, but where the cardioembolic source is highly suspected. Anticoagulation is started in most of these conditions [8]. PFO can also be a source of embolism identified with TEE but is most significant in younger patients (15 years to 49 years) with cryptogenic stroke. PFO can be present in up to 25% of the general population, with the incidence slightly higher in the young population with cryptogenic stroke [9].

With the history of bleeding when on the anticoagulation agent in our patient and the results of previous laboratory evaluation and procedures in the past, how appropriate was it to obtain a hypercoagulable evaluation and TEE during this episode? The importance of a repeat TEE is uncertain in the evaluation of embolism for a known cause and it is inappropriate to use TEE to identify the cause of embolism if no change in the management is anticipated based on the result of TEE [10].

Antiplatelet agents are recommended for patients with ischemic stroke or TIA who have a PFO and are not on anticoagulation [6]. Our patient had known PFO and was not a candidate for anticoagulation because of the previous severe intracranial bleeding while on anticoagulation; therefore, his management would not have changed based on the evaluation he received.

The need for the evaluation of a hypercoagulable state in elderly patients with ischemic stroke or TIA remains unknown [6]. In our patient, who had a complete hypercoagulable evaluation six years earlier, a repeat hypercoagulable evaluation would not contribute to his treatment plan; therefore, he does not satisfy the basic criteria for value-based care.

## Conclusions

An extensively available electronic medical record (EMR) makes the results of previous investigations easily available and gives us the advantage of not repeating tests that do not change the course of the patient's treatment (e.g., hypercoagulable evaluation in our case). As a result, it is eventually helpful in value-based care. Our case is a commonly encountered condition, where the concept of value-based care can save health care costs as well as provide appropriate care to patients.
